# Player Migration and Soccer Performance

**DOI:** 10.3389/fpsyg.2019.00616

**Published:** 2019-03-21

**Authors:** Carlos Lago-Peñas, Santiago Lago-Peñas, Ignacio Lago

**Affiliations:** ^1^Faculty of Education and Sport Sciences, Governance and Economics Research Network, University of Vigo, Pontevedra, Spain; ^2^Faculty of Business and Tourism Studies, Governance and Economics Research Network, University of Vigo, Ourense, Spain; ^3^Department of Political and Social Sciences, Governance and Economics Research Network, Universitat Pompeu Fabra, Barcelona, Spain

**Keywords:** football association, player migration, nation’s soccer performance, endogeneity, globalization, performance analysis

## Abstract

The aim of this study is to examine the relationship between migrating soccer players and the annual ranking of the national teams according to the *World Football Elo Rating*. The sample includes annual data for 243 countries over the period 1994–2018. Migration is captured with the *number of migrating players by country* in the “big-five” leagues. The causal relationship between the two variables is examined by using Granger causality test. Four control variables are included: *the political regime, per capita income, population*, and *regional soccer confederations*. It was hypothesized that (i) the better the ranking of the national teams in the *Elo rating*, the higher the number of migrating players in the “big-five” leagues (shop-window hypotheses) and that (ii) while the shop-window effect takes place in the short-run, the annual *Elo rating* of a national team is positively affected by expatriate players in the medium or long-run, but not in the short-run (blending hypotheses). The results shed light on two crucial issues. First, causality mainly goes from national soccer performance to migrating soccer players rather than the other way around. Second, the timing of the two effects is quite different. While those players giving an outstanding performance when their national team is doing well are immediately bought by clubs from more highly ranked leagues (the shop-window effect), it takes at least 4 years for the additional skills acquired by migrated players to have a positive effect on the national soccer performance (the blending effect).

## Introduction

Globalization —the process fueled by, and resulting in, increasing cross-border flows of goods, services, money, people, information, and culture (Held et al., 1999, p. 16)— has dramatically affected domestic societies over the last decades and stimulated an intense research in economics, sociology, political science or anthropology (Berger, 2000; Guillén, 2001; Steger, 2017).

Soccer is not isolated from globalization. Free circulation of players has substantially increased during the last 25 years, as limits on the number of foreign players in the European leagues have been lifted and clubs become more commercially minded (Milanovic, 2005). According to Gelade and Dobson (2007: 250), between 2000 and 2005 the 40.9% of the players representing their country in international competition played club soccer abroad, and the 86.2% of them played in a country with a higher FIFA ranking. More recently, as of May 1, 2017, 12,051 expatriate footballers were recorded in the 2,120 clubs competing in 137 leagues of 93 national associations worldwide. On average, a team has 5.7 expatriate footballers, meaning that foreign players represent 21.6% of the average squad (Poli et al., 2017). In 2018, the proportion of expatriate players in a sample of 31 top division leagues of UEFA members association has increased to a record level of 41.5% (Poli et al., 2018). In addition, they are increasingly important in their teams. According to the most recent available data (November 2018), the percentage of minutes played by foreign players is 64.7% in the English Premier League (EPL), 61% in the Italian Serie A, 51.5% in the German Bundesliga, 39% in the Spanish Liga and 37.3% in the French Ligue 1 (Poli et al., 2018).

Interestingly, existing research is overwhelmingly focused on the impact of soccer players’ migration to foreign clubs on the international soccer performance of their country of origin (Gásquez and Royuela, 2016). Conventional (although not universal) wisdom establishes that soccer players’ migration is positively correlated with countries’ international soccer performance, particularly in poorer countries or countries with lower-quality soccer clubs. Clearly, the greater the number of good players formed in a country, the better the performance of the national soccer team. The mechanism accounting for this positive effect of soccer player migration is the additional skills acquired by migrating players in top foreign leagues (Berlinschi et al., 2013). More specifically, expatriate players bring additional resources of experience, skill, and fitness to their national teams (Gelade and Dobson, 2007). This is what we call the *blending* argument. However, in (rich) countries with high-quality domestic leagues, the expected effect of migration is not clear (Leeds and Leeds, 2009; Yamamura, 2009; Berlinschi et al., 2013; Allan and Moffat, 2014). For a less optimistic view about the effect of football player migration on national team performance, see Frick (2007) or Maguire (2008).

Surprisingly, how national team performance affects the migration of soccer players remains largely unexplored. Players’ migration to foreign clubs should increase when national teams do well. Expatriate soccer players mainly move from countries with lower-quality domestic leagues to the major leagues in Europe, primarily the “big-five” European leagues (English Premier League, Italian Serie A. Spanish La Liga, French Ligue 1, and German Bundesliga) (Deloitte, 1997–2018). According to the data compiled by Poli (2010), in 1995–1996 there were 463 expatriate players in the “big-five” leagues or 20.2% of the total number of players in the “big-five” leagues, while in 2008–2009 there were 1,107 expatriates, accounting for 42.6% of players. The “big-five” leagues are conventionally studied separately from the rest of leagues due to the high aggregated market value of their teams (see, for instance, Frick, 2009; Poli, 2010; Kuper and Szymanski, 2014). Migration should be expected to increase when players from countries with lower-quality domestic leagues are in the spotlight - that is, immediately after a World Cup. In fact, it has been demonstrated that players who have recently participated in the World Cup appear to benefit from a double effect, both by raising player salaries paid by clubs and by helping players secure transitions to more highly - ranked teams (Simmons and Deutscher, 2012; Kuper and Szymanski, 2014). This is what we call the *shop-window* argument.

Despite the shop-window argument, reverse causality has been addressed very differently when testing the blending argument. Most existing empirical research simply ignores it. For instance, Gelade and Dobson (2007) rely on cross-section data for 201 countries and show that the percentage of expatriate players in the national teams positively affects the average country’s FIFA rating over the 2000–2005 period. Similarly, using cross-sectional data for 170 countries in 2010, 2011, and 2012, Allan and Moffat (2014) found that player emigration has a positive impact on the performance of the national soccer team. On the contrary, when explaining the national team performance in 2010 in 202 countries, Berlinschi et al. (2013) take reverse causality into account. They find that migration of national team players improves international soccer performance. Reverse causality between national team performance and population and migration is addressed using population size as a proxy for each country’s talent pool and performing instrumental variable estimations. Finally, the endogeneity problem is taken very seriously in the time-series analysis conducted by Vasilakis (2017). Using data from nine World Cup years (1978–2010) in 65 countries and Two-Stage Least Squares (2SLS), he shows that the total number of talented players weighted by the score of their employment league is a key determinant of national team performance.

In sum, the aim of this study is to examine the relationship between migrating soccer players and the annual ranking of the national teams according to the *World Football Elo Rating*. To the best of our knowledge, no studies have explored whether the causal relationship between migrating soccer players and national soccer performance is bidirectional. It was hypothesized that (i) the better the ranking of the national teams in the *Elo rating*, the higher the number of migrating players in the “big-five” leagues (shop-window hypotheses) and that (ii) while the shop-window effect takes place in the short-run, the annual *Elo rating* of a national team is positively affected by expatriate players in the medium or long-run, but not in the short-run (blending hypotheses).

## Materials and Methods

### Sample

To examine the causal relationship between the annual ranking of the national teams according to the *World Football Elo Rating* and the migration of soccer players, data were collected from 243 countries for which annual data on the two variables are available over the period 1994–2018. The sources are www.eloratings.net and www.transfermarkt.com. In order to control for the impact of the Bosman transfer ruling, a sectorial liberalization shock to football labor markets that banned quotas on the number of foreigners playing for a club (Frick, 2009; Binder and Findlay, 2012), we start in 1994.

### Variables

National soccer performance and migrating soccer players are measured using the *Elo rating* and the *number of migrating players* by country in the “big-five” leagues, respectively. First, the *World Football Elo Rating* is a ranking system for men’s national association soccer teams published by www.eloratings.net and increasingly used in the soccer literature (e.g., Binder and Findlay, 2012 or Gásquez and Royuela, 2016). *Elo Ratings* are based on the work of Arpad Elo. The Ratings were developed for chess but they have been adapted for other games, including soccer. In these Ratings, there is: a weighting for the kind of match played; an adjustment for home team advantage and an adjustment for goal difference in the match result. The formula used to calculate the *Elo Rating* is Rn = Ro + K × (W – We), in which: Rn is the new rating; Ro is the old (pre-match) rating; K is the weight constant for the tournament played; K is then adjusted for the goal difference in the game. It is increased by half if a game is won by two goals, by 3/4 if a game is won by three goals, and by 3/4+ (N-3)/8 if the game is won by four or more goals, where N is the goal difference; W is the result of the game (1 for a win, 0.5 for a draw, and 0 for a loss); We is the expected result from this formula. We = 1/[10(-dr/400)+ 1] in which dr equals the difference in ratings plus 100 points for a team playing at home.

As explained by Gásquez and Royuela (2016: 8), the *Elo rating* solves the methodological problems of the FIFA rating: the confederation effect, the high volatility among the rankings of the top 10 teams and the limited information it employs (i.e., exclusively whether the team wins, loses, or draws the match). The *Elo rating* uses a low volatility index (an index that has more memory present), does not depend on the confederation to which a national team belongs, and incorporates more information, in particular, the expected and goal difference in the game. Finally, the FIFA ranking underwent methodological changes in 1999 and 2006, while the *Elo rating* has not. Given that the period we are covering in our empirical analysis is 1994–2018, the *Elo rating* allows comparisons over time. The *Elo rating* in our sample ranges from a minimum rating of 354 points for Eastern Samoa in 2007, 2008, 2009, and 2010 to a maximum value of 2,182 points for Brazil in 1997. The mean value is 1,334 ± 365 for all the countries and years.

The number of migrating players has been calculated as the raw number of foreign players by country in the “big-five” leagues. The mechanism driving the relationship between a national team’s performance and the migration of soccer players is that expatriate soccer players move from countries with lower-quality domestic leagues to countries with high-quality domestic leagues. However, an increasing number of players are moving in the opposition direction. In particular, Major Soccer League (MLS) in the United States and Canada has become a destination for many aging stars. When focusing on the migrating of players moving to the “big-five” leagues, the bottom-up movement in terms of the quality of domestic leagues is clear. National players in their domestic leagues do not count as migrating players. Our assumption is that is that if expatriate players are moving to higher-quality domestic leagues (i.e., expatriate players are better than the average player in their origin countries), all or most of them should play in their national teams. Additionally, as there is no available information for all expatriate players in all the domestic leagues in the world, it is not possible to determine in all cases whether a player is moving to a better or a worse domestic league. The source is www.transfermarkt.com. The *number of migrating players* in our sample goes from 0 in many countries and years to 157 in Brazil in 2008. The mean is 5.2 ± 12.8 for all the countries and years. The descriptive statistics of these two key variables are displayed in Table 1.

**Table 1:** Descriptive statistics.

	*ELO rating*	*The number of migrating players*
Mean	1334	5.2
Median	1368	0
Standard deviation	365	12.8
Maximum	2182	157
Minimum	354	0


*5760 common observations*.

We also included some conventional controls when explaining soccer success (Gásquez and Royuela, 2016). In particular: (i) the *political regime* (i.e., whether the country is a democracy, 1, or a non-democracy, 0) using the regime classification by Cheibub et al. (2010); (ii) *per capita incom*e in constant dollars (data retrieved from https://data.worldbank.org/indicator/NY.GDP.PCAP.KD; (iii) *population* (data retrieved from https://data.worldbank.org/indicator/sp.pop.totl); and (iv) *regional football confederations* (AFC, CAF, CONCACAF, CONMEBOL, OFC or UEFA).

### Statistical Analysis

The empirical analysis proceeds in three steps. The first step consists of examining the *Data Generator Process* (DGP) of *Elo rating* and *the number of migrating players* to determine whether the variables are stationary or integrated and, therefore, whether the empirical analysis has to be performed in levels or first differences. Two panel unit root tests, the Levin-Lin-Chu (Levin et al., 2002) and the Im-Pesaran-Shin (Im et al., 2003) tests have been employed. While the former assumes common slopes, the second computes individual slopes.

In the second step, the analysis is focused on the link between migrating soccer players and national soccer performance. Using the Granger causality test, we assess whether this link is unidirectional or bidirectional. Testing causality through the Granger (1969) approach involves determining whether lagged information on a variable Y provides any statistically significant information about a variable X in the presence of lagged values of X. If not, Y does not Granger-cause X. We test whether causality runs from the migration of soccer players to national soccer performance, as the blending hypothesis argues, or whether it runs from national soccer performance to the migration of soccer players, as the shop-window hypothesis argues. We have run the test using a number of lags, going from 2 (i.e., short-run) to 6 (i.e., long-run). We start from lag 2 instead of lag 1 in order to avoid a bias due to the omission of relevant independent variables. As the first two lags of the endogenous variable are statistically significant for the two dependent variables, using only one generates a bias in the analysis. The results do not change appreciably when increasing the number of lags, but reduces the number of observations.

Finally, the third step consists of quantifying the interplay between *Elo rating* and *the number of migrating players* using Vector Autoregression (VAR) models. In order to interpret the results intuitively, standard impulse-response figures are employed (Lütkepohl, 2008). When estimating the VAR models, we included the four controls in addition to the lags of *Elo rating* and *number of migrating players:* (1) whether the country is a *Democracy* or not, (2) *per capita GDP*, (3) *population*, and (4) *regional soccer confederations*. Only *Democracy* was statistically significant (at the 1% level) when explaining the number of expatriate players. All other things being equal, players’ migration to foreign clubs increases when the country of origin is a democracy. Accordingly, *Democracy* has been included in the final specification when explaining *the number of migrating players*. The average of *Democracy* is 0.52. Individual fixed effects are excluded in order to avoid multicollinearity with *Democracy*. All statistical analyses were performed using Eviews for Windows, version 10.0.

## Results

**FIGURE 1:**
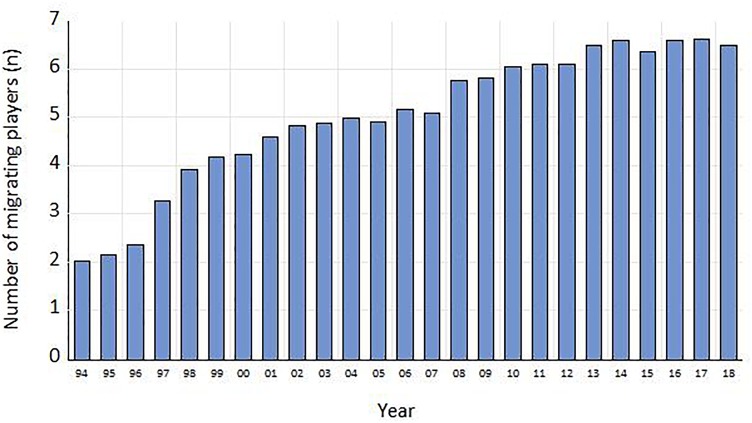
Evolution of the average number of migrating players over time per country.

The average number of migrating players per country in the “big-five” leagues in the period 1994–2018 is presented in Figure 1. Over the 25-year period, the number of expatriate footballers has multiplied by 3, from 2 in 1994 to more than 6 after 2012.

The results of the two panel unit root tests are displayed in Table 2. For both variables the hypothesis of unit roots is rejected at the 1 percent level using both the Levin-Lin-Chu and the Im-Pesaran-Shin tests. Accordingly, the variables are measured in levels.

**Table 2:** Panel unit root tests.

	*Elo rating*	*Number of migrating players*
Levin-Lin-Chu test (*p*-value)	0.0006^∗∗^	0.0028^∗∗^
Im-Pesaran-Shin test (*p*-value)	0.0003^∗∗^	0.00001^∗∗^


*Tests include individual intercepts. ^∗∗^*p* < 0.01*.

As can be seen in Table 3, the results of the Granger causality tests strongly support the shop-window hypothesis. The null hypothesis that *Elo rating* does not cause the number of migrating players is rejected at the 1% level in all cases. However, the blending hypothesis is only supported when using four or more lags, at the *p* < 0.05 level or less. When considering 2 or 3 lags, the hypothesis is not supported. In other words, it takes at least 4 years before an improvement in national soccer performance, thanks to the additional skills acquired by migrated players in top foreign leagues, becomes evident.

**Table 3:** Causality tests for different lags structures (*p*-values are shown).

	2 lags	3 lags	4 lags	5 lags	6 lags
*Elo rating does not cause the number of migrating players*	0.0005^∗∗^	0.0002^∗∗^	0.0049^∗∗^	0.0026^∗∗^	0.0099^∗∗^
*The number of migrating players does not cause Elo rating*	0.101	0.254	0.0357^∗^	0.0211^∗^	0.0067^∗∗^


*Tests include individual intercepts. According to the diagnostic tests, fixed effects are highly significant, while the time trend is not. Although autoregressive models with fixed effects lead to biased parameter estimates (Nickell, 1981), the bias is of order 1/T, where T is the number of time periods Accordingly, when T is equal or greater than 20, as in our case, the problem tends to fade out. Additionally, according to the Monte Carlo evidence provided by Beck and Katz (2011), the usual corrections for this bias do not perform better than the Least Squares Dummy Variables (LSDV) when T is greater than 20. ^∗^*p* < 0.05; ^∗∗^*p* < 0.01*.

**FIGURE 2:**
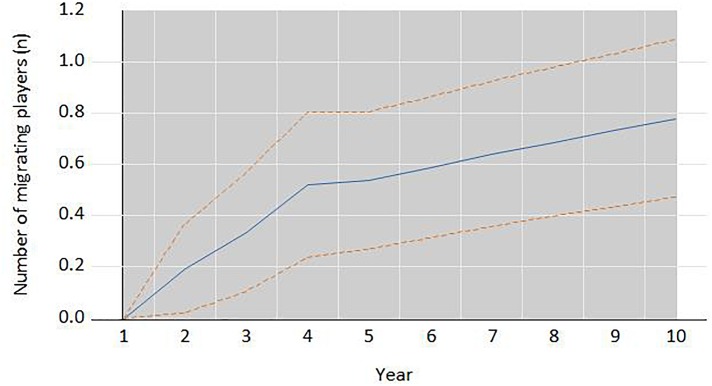
Response of the number of migrating players per country to a shock of +100 points in the *ELO rating* in year 1. The dotes lines are the whole plus/minus two standard error bands about the impulse responses is in positive ground. The shaded area in gray indicates statistical significance at 5% or less.

**FIGURE 3:**
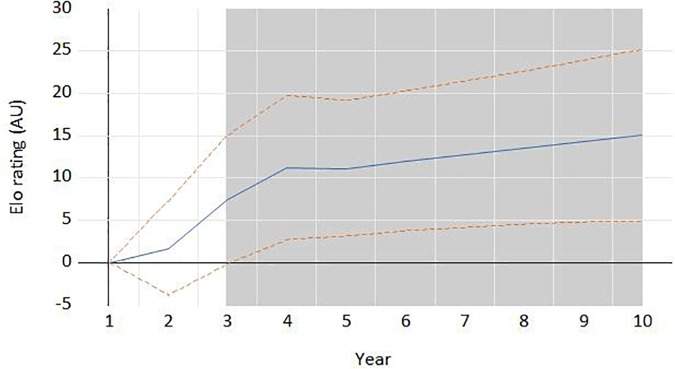
Response of the *ELO rating* to an increase of 10 migrating players per country in year 1. The dotes lines are the whole plus/minus two standard error bands about the impulse responses is in positive ground. The shaded area in gray indicates statistical significance at 5% or less.

Figure 2 and 3, respectively, show the impulse-response of the *Elo rating* and the number of migrating players to shocks in the other variable. We have simulated the effect of two external shocks: an increase of 100 points in the *Elo rating* and an increase of 10 migrant players. As can be seen in Figure 2, the shock to the *Elo rating* has a positive and statistically significant effect on migrating players since the first year (the whole plus/minus two standard error bands about the impulse responses is in positive ground). There is evidence of a very steep curve, particularly in the first four years. For instance, an increase of 100 points in the *Elo rating* in a given year generates and average increase of 0.2 migrating players in the next year. However, the Figure 3 shows that the effect of the number of migrating players on the *ELO rating* is only statistically significant in the third and following years, but not in the first two.

## Discussion

This article has examined the reverse causality between migrating soccer players and national soccer performance. The proportion of expatriate footballers has increased markedly in the last 25 years (Poli et al., 2017, 2018). Existing research is overwhelmingly focused on what we have labeled the blending hypothesis, that is, that soccer players’ migration is positively correlated with their nation’s soccer performance in international competition, especially in poorer countries or countries with lower-quality soccer clubs. Interestingly, how a national team’s performance affects the migration of soccer players (i.e., the shop-window hypothesis) has not been examined.

Our empirical analysis sheds light on two crucial issues. First, the causal relationship mainly goes from national team performance to migrating soccer players, rather than the other way around. Second, the timing of the two effects is quite different. While the shop-window effect - the better the ranking of the national teams in the *Elo rating*, the higher the number of migrating players in the “big-five” leagues - (Simmons and Deutscher, 2012; Kuper and Szymanski, 2014) takes place in the short-run, the blending effect - the annual *Elo rating* of a national team is positively affected by expatriate players - (Gelade and Dobson, 2007; Leeds and Leeds, 2009; Yamamura, 2009; Berlinschi et al., 2013; Allan and Moffat, 2014) is only observed in the medium or long-run. The mechanisms driving the diverging timing of the effects are as follows: on the one hand, when the *Elo rating* of a national team increases (which is particularly significant in World Cup years), their players are immediately bought by clubs from more highly ranked leagues (Simmons and Deutscher, 2012; Kuper and Szymanski, 2014). On the other hand, the performance of migrating players may require several years of experience to improve, especially for players from leagues of lower quality. As a result, it takes at least 4 year before the positive effect of the additional skills acquired by migrated players are visible in the national team’s performance. Thus, the blending argument is partially confirmed.

This finding is in line with the empirical and anecdotal evidence provided by Kuper and Szymanski (2014). According to them, “the worst moment to buy a player is in the summer when he’s just done well at a big tournament. Everyone in the transfer market has seen how good the player is, but he is also exhausted and quite likely sated with success. As Fergusson admitted after retiring from Manchester United: I was always wary of buying players on the back of good tournament performances. I did it at the 1996 European Championship, which prompted me to move for Jordi Cruyff and Karel Poborsky. Both had excellent runs in that tournament but I didn’t receive the kind of value their countries did that summer. They weren’t bad buys, but sometimes players get themselves motivated and prepared for World Cups and European Championships and after that there can be a leveling off [included in Kuper and Szymanski (2014)].”

The crucial implication of our analysis is that endogeneity is a serious problem when examining the relationship between migrating soccer players and national soccer performance. By ignoring the feedback effect, the correct inference plus a bias factor is estimated. When a national team’s soccer performance is the dependent variable, endogeneity already emerges when using the values of migrating soccer players from the previous year. When migrating soccer players is the outcome, endogeneity is an issue when using lagged values of national team’s performance. We urge researchers to take endogeneity very seriously in empirical analyses.

These findings may help coaches and managers to better understand how the success of national teams affects migration in elite soccer and may have the potential to assist in decisions such as, for example, when a new contract should be signed, the duration of the contract or when to replace or transfer a player depending on the moment of season. For example, the worst moment to buy a player is in summer when s/he’s done well at an international tournament. On the other hand, the selling teams should not transfer a player before a big tournament if the market price is not high enough. The player can revalue after a good performance in a major national soccer event (i.e., World Cup or Continental Soccer Championship).

Concerning the limitations of the current study, some aspects should be highlighted. Future research should move from the nation/aggregated level to the player/individual one. Other variables such as the age of the players, their playing position, the quality of the receiving clubs or the number of played minutes should be included in future studies, given that they can affect their performance in the receiving countries’ leagues and the additional skills they bring to their national teams. The number of migrating players in other leagues should also considered. In addition, the manager immigration variable should be also included in future studies (Allan and Moffat, 2014).

## Conclusion

In conclusion, the results of the Granger causality tests support the shop-window hypothesis when examining the relationship between migrating soccer players and national soccer performance. Those players with an outstanding performance when the national team is doing well are immediately bought by top clubs. However, the blending hypothesis is only confirmed (on average) 4 years after players’ migration: national team performance is positively affected by expatriate players in the medium or long-run, but not in the short-run.

## Data Availability

The datasets generated for this study are available on request to the corresponding author.

## Author Contributions

All authors listed have made a substantial, direct and intellectual contribution to the work, and approved it for publication.

## Conflict of Interest Statement

The authors declare that the research was conducted in the absence of any commercial or financial relationships that could be construed as a potential conflict of interest.
